# Factors that influence rheumatologists’ anti-tumor necrosis factor alpha prescribing decisions: a qualitative study

**DOI:** 10.1186/s41927-019-0097-0

**Published:** 2019-12-19

**Authors:** Sean P. Gavan, Gavin Daker-White, Katherine Payne, Anne Barton

**Affiliations:** 1 Manchester Centre for Health Economics, Division of Population Health, Health Services Research and Primary Care, School of Health Sciences, Faculty of Biology, Medicine and Health, The University of Manchester, Manchester Academic Health Science Centre, Oxford Road, Manchester, M13 9PL UK; 2 NIHR Manchester Biomedical Research Centre, Manchester University NHS Foundation Trust, Manchester Academic Health Science Centre, Manchester, UK; 3 NIHR Greater Manchester Patient Safety Translational Research Centre, Division of Population Health, Health Services Research and Primary Care, School of Health Sciences, Faculty of Biology, Medicine and Health, The University of Manchester, Manchester Academic Health Science Centre, Oxford Road, Manchester, M13 9PL UK; 4 Arthritis Research UK Centre for Genetics and Genomics, Centre for Musculoskeletal Research, Division of Musculoskeletal and Dermatological Sciences, School of Biological Sciences, Faculty of Biology, Medicine and Health, The University of Manchester, Manchester Academic Health Science Centre, Oxford Road, Manchester, M13 9PL UK

**Keywords:** Clinical recommendations, DAS28, Health economics, NICE, Prescribing behaviour, Qualitative study, Regional variation, Resource use, Rheumatoid arthritis

## Abstract

**Background:**

Treatment decisions for any disease are usually informed by reference to published clinical guidelines or recommendations. These recommendations can be developed to improve the relative cost-effectiveness of health care and to reduce regional variation in clinical practice. Anti-tumor necrosis factor alpha (anti-TNF) treatments are prescribed for people with rheumatoid arthritis according to specific recommendations by the National Institute for Health and Care Excellence in England. Evidence of regional variation in clinical practice for rheumatoid arthritis may indicate that different factors have an influence on routine prescribing decisions. The aim of this study was to understand the factors that influence rheumatologists’ decisions when prescribing anti-TNF treatments for people with rheumatoid arthritis in England.

**Methods:**

Semi-structured one-to-one telephone interviews were performed with senior rheumatologists in different regions across England. The interview schedule addressed recommendations by the National Institute for Health and Care Excellence, prescribing behavior, and perceptions of anti-TNF treatments. Interviews were recorded digitally, transcribed verbatim, and anonymized. Data were analyzed by thematic framework analysis that comprised six stages (familiarization; coding; developing the framework; applying the framework; generating the matrix; interpretation).

**Results:**

Eleven rheumatologists (regional distribution - north 36%; midlands: 36%; south: 27%) participated (response rate: 24% of the sampling frame). The mean duration of the interviews was thirty minutes (range: 16 to 56 min). Thirteen factors that influenced anti-TNF prescribing decisions were categorized by three nested primary themes; specific influences were defined as subthemes: (i) External Environment Influences (National Institute for Health and Care Excellence Recommendations; Clinical Commissioning Groups; Cost Pressures; Published Clinical Evidence; Colleagues in Different Hospitals; Pharmaceutical Industry); (ii) Internal Hospital Influences (Systems to Promote Compliance with Clinical Recommendations; Internal Treatment Pathways; Hospital Culture); (iii) Individual-level Influences (Patient Influence; Clinical Autonomy; Consultant Experience; Perception of Disease Activity Score-28 (DAS28) Outcome).

**Conclusions:**

Factors that influenced anti-TNF prescribing decisions were multifaceted, seemed to vary by region, and may facilitate divergence from published clinical recommendations. Strategic behavior appeared to illustrate a conflict between uniform treatment recommendations and clinical autonomy. These influences may contribute to understanding sources of regional variation in clinical practice for rheumatoid arthritis.

## Background

Structured guidelines and recommendations are used to inform routine clinical decision-making in health care systems around the world [1]. The National Institute for Health and Care Excellence (NICE) publish national recommendations for patients in England [2] and have influenced the process for producing evidence-based recommendations internationally [3]. Health care professionals will be aware of the specific recommendations for treatments made during the NICE technology appraisal program because, for the majority of cases, their implementation is mandatory within 3 months [2]. For example, the NICE technology appraisal program has produced recommendations for prescribing biologic therapies to people with rheumatoid arthritis (RA) that are naïve to conventional disease-modifying antirheumatic drugs (cDMARDs), after experiencing inadequate response to methotrexate, and after experiencing inadequate response to a previous biologic agent [4, 5]. Clinicians and health care professionals, in practice, are able to interpret the applicability of specific NICE recommendations in the context of each patient [6, 7].

Two objectives of NICE recommendations are (i) to facilitate a cost-effective use of limited resources for health care and (ii) to minimize regional variation in health care [8]. National clinical audits for RA and early inflammatory arthritis, published by the British Society for Rheumatology in 2015 and 2016, illustrated regional variation in practice across seven NICE quality standards [9, 10]. For example, in 2015/16, 60% of patients in the midlands/eastern regions of England commenced cDMARDs within 6 weeks of referral compared with 73% of patients in the southern region [10]. Tugnet et al. [11] surveyed 311 patients with RA from 19 rheumatology units in a specific region and found between-hospital variation in the uptake of the same quality standards. Similarly, Blake et al. [12] found that only 65% of patients with RA who changed treatment between two biologic agents were compliant with NICE recommendations; the degree of compliance also varied between the eighteen rheumatology units from which these data were collected. The factors that influenced this variation were unclear given the presence of common national treatment recommendations.

Biologic treatments are embedded within the pathway of care for RA [13]; in 2018, there are five biologic anti-TNF agents, comprising reference and biosimilar therapies, with marketing authorization for RA (adalimumab, certolizumab pegol, etanercept, golimumab, and infliximab) [14]. Meta-analyses have demonstrated that, on average, the effectiveness of these five anti-TNF agents can be viewed to be comparable [15]. In 2016, NICE recommended anti-TNF agents for people with severe active RA (Disease Activity Score-28 Joint Count (DAS28) ≥ 5.1) who had failed to respond to cDMARDs [5]. The specific recommendation made by NICE with respect to the choice of first-line anti-TNF was:

*“1.5. Start treatment with the least expensive drug (taking into account administration costs, dose needed and product price per dose)”* [5].

Rheumatologists in England have access to the cost of treatments because they are reported within the British National Formulary which is made available for free (hard-copy and online access) to health care professionals [16]. The cost of a biologic agent may also be negotiated at the regional-level which will be communicated to prescribers [17]. The decision to prescribe a particular anti-TNF agent may be more complex in practice than the cost-minimization strategy recommended by NICE. Qualitative methods are being used increasingly in rheumatology research to obtain a deeper understanding of complex phenomena [18, 19]. These methods have been used extensively to understand patients’ beliefs and experiences about their own treatments and disease [20–22]. Qualitative studies have been used previously to explore the influences on biologic prescribing decisions for RA in Sweden [23] and Ireland [24]. No study has used these methods to explore the factors that may influence anti-TNF prescribing decisions for RA in England; a greater understanding of this phenomenon may contribute to identifying drivers of the observed regional variation in care in the presence of uniform national recommendations. The aim of this study, therefore, was to understand the factors that influence rheumatologists’ decisions when prescribing anti-TNF treatments for people with RA in England.

## Methods

Semi-structured one-to-one telephone interviews were conducted with senior clinical rheumatologists from different regions across England. The study was performed and reported according to the 2014 Standards for Reporting Qualitative Research [25].

### Target population and sample

The target population comprised senior rheumatologists who had experience of treating RA. This target population was chosen because they were expected to have good working knowledge of the key NICE recommendations that guide practice and extensive experience of using anti-TNF agents to manage people with RA. A purposive sample was recruited by occupation using the list of principal investigators from the ‘Biologics in Rheumatoid Arthritis Genetics and Genomics Study Syndicate’ as the sampling frame. The role of principal investigator was indicative of seniority and extensive experience of managing RA using biologic anti-TNF agents. Individuals in the sampling frame were based at different hospitals across the country (one individual per hospital). An identical recruitment email and participant information sheet was sent to all rheumatologists in December 2014 and March 2015.

### Data collection

Data were collected using semi-structured interviews with open-ended questions guided by an interview schedule that addressed six topics (Table 1). A pilot interview was performed with a clinical research fellow, experienced in treating people with RA, to ensure that the interview questions and structure were suitable. Additional interview questions were posed according to the responses of preceding participants, consistent with the grounded theory method of qualitative research [26]. Recruitment continued until all individuals in the sampling frame who agreed to participate were interviewed.

**Table 1:** Six Topics Addressed by the Interview Schedule

Topic	Description
Topic 1	Interpretation of NICE recommendations
Topic 2	Procedures to ensure compliance with NICE recommendations
Topic 3	Assessing the suitability of anti-TNF therapy
Topic 4	The choice of first anti-TNF therapy
Topic 5	Prescribing decisions following the failure of an anti-TNF
Topic 6	Beliefs about the anti-TNF agents recommended by NICE

NICE: National Institute for Health and Care Excellence

Participants were interviewed over the telephone to increase the feasibility of collecting data from clinicians distributed across the country [27]. Rheumatologists who provided consent scheduled their interview at a date and time convenient for them and the interviewer. Telephone interviews were recorded by a digital audio recorder and the interview content was transcribed verbatim [28]. The audio recording of each interview was replayed after transcription to ensure data integrity and congruence between the audio and transcript. Transcripts were anonymized by removing references to names and locations. All interviews and transcriptions were performed by one author (SPG) who had undertaken training in the collection and analysis of qualitative data.

### Data analysis

The interview transcripts were analyzed using thematic framework analysis that comprised six stages [29–31]. This method of analysis identified and tabulated common themes from the range of views provided during the rheumatologists’ interviews. The six stages are now described:

#### Stage 1: familiarization

The research team became immersed in the data by reading each transcript actively to identify initial patterns of response within and between each participant [30].

#### Stage 2: coding

The lines of each transcript were categorized with a specific code. Each code was a descriptive label that conveyed a meaning in relation to the research aim [30]. For example, codes may have referred to explicit statements within the transcripts or to the emotions conveyed by a participant [31]. Coding was systematic and thorough which enabled excerpts of the transcripts to be compared across the sample [30, 31]. Coding was performed across all transcripts by the lead author; supplementary coding was performed by two other authors to enhance the trustworthiness of the analysis.

#### Stage 3: developing the framework

Codes within and between transcripts were grouped together according to their similarity to form themes. A theme comprised a distinct set of codes that shared a common element and reflected a pattern of responses across sample [30].

#### Stage 4: applying the framework

The interview transcripts were analyzed continually during data collection. Themes and codes that were identified within earlier transcripts were applied to subsequent transcripts. The analytic framework was refined if excerpts of subsequent transcripts did not relate to a theme or code that was identified earlier [29].

#### Stage 5: generating the framework matrix

A separate matrix (a row for each participant and a column for each code) was created for each theme [31]. Cells within each matrix were populated by charting data from the transcripts of each participant [29, 31].

#### Stage 6: interpretation

The responses of all participants were compared for each code and theme after all data were charted to the framework matrix [29]. The results were reported by theme using a narrative synthesis of responses with supporting quotations. The dispersion of themes that were identified in each transcript was evaluated after all interviews had been conducted to interpret the extent of thematic saturation within the sample.

### Ethics

This research protocol was approved by The University of Manchester Research Ethics Committee 2 (reference number: 14147). All participants contributed voluntarily and received no financial compensation. Written informed consent and agreement to the publication of anonymous quotations was obtained from all participants.

## Results

**Fig. 1:**
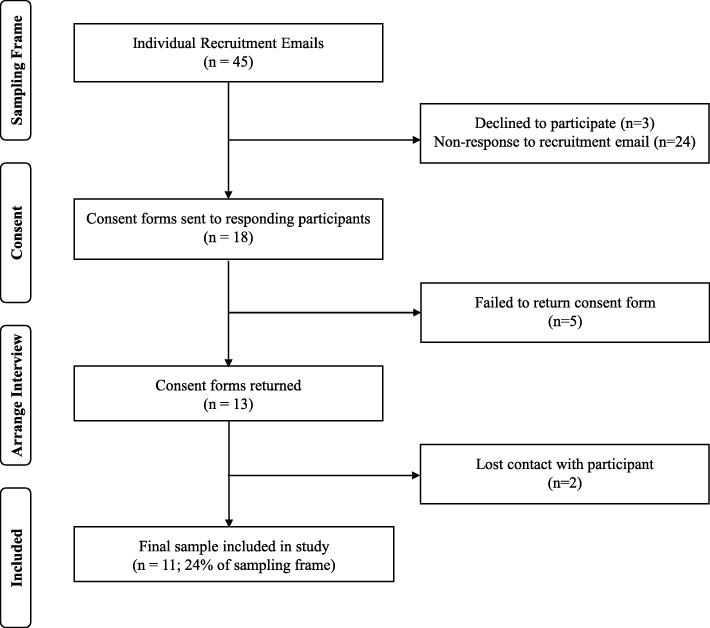
Flow Diagram of Participant Recruitment

Figure 1 presents a flow-diagram to illustrate participant recruitment. Forty-five rheumatologist were invited to participate; eleven rheumatologists (male: 82%; female: 18%) were interviewed between January and September 2015 (response rate: 24%). The rheumatologists in the sample were distributed across England (north: 36%; midlands: 36%; south: 27%). The mean duration of the interviews was 30 min (range: 16 to 56 min).

**Fig. 2:**
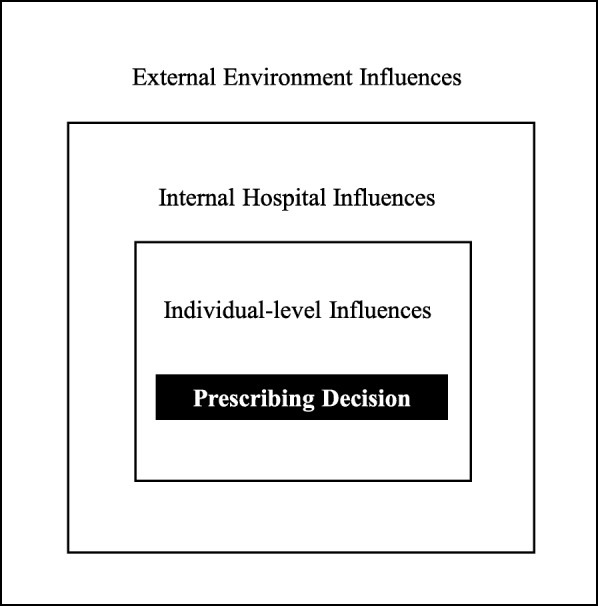
Illustration of Factors that Influence Prescribing Decisions as Three Nested Primary Themes

**Fig. 3:**
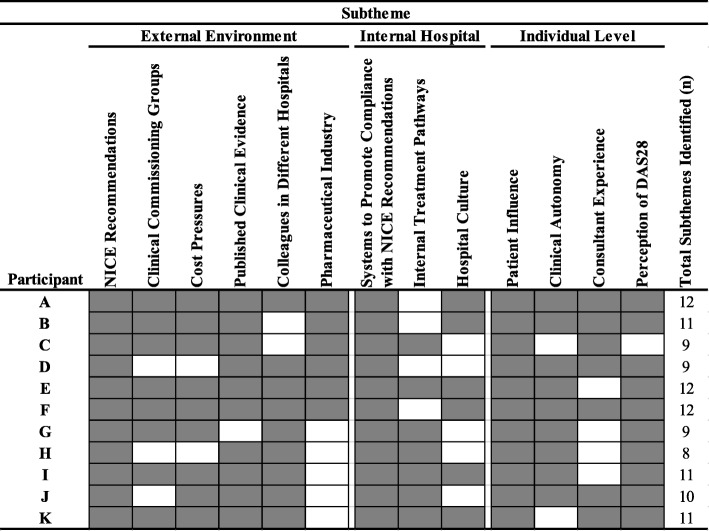
Distribution of Themes and Subthemes Identified in each Transcript. Each shaded area denotes that the specific subtheme was identified in the corresponding rheumatologist’s transcript

The rheumatologists’ responses indicated that anti-TNF prescribing decisions were made within a system characterized by three nested primary themes of influential factors (Fig. 2): (i) the wider context in which a hospital functioned (*External Environment Influences*); (ii) within a hospital (*Internal Hospital Influences*); and (iii) the factors closest to the rheumatologist (*Individual-level Influences*). Thirteen specific influences were classified as subthemes within each primary theme (Table 2). Figure 3 illustrates the dispersion of subthemes reported by each rheumatologist. A mean of 10 subthemes (range: 8 to 12 subthemes) were identified in each transcript. Unique subthemes were not described by the rheumatologists after the third interview which was indicative of thematic saturation in the sample. The primary themes and subthemes are now described with supporting quotations from the rheumatologists labeled anonymously using letters A to K.

**Table 2:** Primary Themes and Subthemes of Factors that Influence Anti-TNF Prescribing Decisions

Primary Theme	Subtheme
External Environment	NICE Recommendations Clinical Commissioning Groups Cost Pressures Published Clinical Evidence Colleagues in Different Hospitals Pharmaceutical Industry
Internal Hospital	Systems to Promote Compliance with NICE Recommendations Internal Treatment Pathways Hospital Culture
Individual-level	Patient Influence Clinical Autonomy Consultant Experience Perception of DAS28

DAS28: Disease Activity Score-28 Joints; NICE: National Institute for Health and Care Excellence

### External environment influences

Six influences identified from the interview transcripts that were categorized by the external environment are now described.

#### NICE recommendations

The rheumatologists framed prescribing decisions around the recommendations made by NICE. These recommendations were perceived as suitable for most patients with RA but were occasionally difficult to interpret, in particular, when the eligibility criteria for anti-TNF therapy were not met.

**J**: *“ … NICE guidance tends to provide a linear algorithm of the way to go and … if you don’t end up on that linear algorithm, then … it’s just not clear what’s allowed”.*

NICE recommendations were interpreted differently across the sample, characterized by two disparate positions (flexible and inflexible).

**H**: *“ … almost all NICE guidance is open to interpretation … Guidance is guidance. It’s not … the law that has to be followed, otherwise you go to jail or something”.*

**I**: *“[NICE guidance] is not open enough, in my view. It should be more open”.*

#### Clinical commissioning groups

Health care services in England are commissioned regionally by clinical commissioning groups (CCGs) [32]. The rheumatologists perceived their CCG as an enforcer of NICE recommendations. Some rheumatologists described that their CCG (typically those in a worse financial position) imposed the choice of first-line anti-TNF which restricted clinical autonomy.

**K**: *“Our CCG is a bit strapped for cash so we are not allowed to deviate one iota [from NICE recommendations]”.*

**G**: *“I should say, it’s not a particularly popular decision with the clinicians … because we want to have free choice of biologics”.*

#### Cost pressures

Most rheumatologists in the sample suggested that cost had limited influence on their prescribing decisions, despite NICE recommending the lowest-cost anti-TNF in routine practice.

**A**: *“I think there’s lip service paid to total acquisition cost”.*

However, a sense of social duty was expressed by others in the sample when considering the sustainability of high-cost treatments in the NHS.

**F**: *“I think we should be obliged, as clinicians, to consider costs with every treatment decision we make … Every anti-TNF drug we start takes money out of the health service that could be used for other purposes or other patients”.*

The sample expressed difficulty in making cost-savings; two strategies to mitigate the high cost of treatments were to (i) undertake regional price negotiations of anti-TNF therapies and (ii) to engage in research studies where the experimental treatment was received free-of-charge. The implications of failing to reduce costs also influenced treatment decisions as illustrated by this quote:

**K**: *“We prefer to use a … biosimilar than sack nurses, quite honestly”.*

#### Published clinical evidence

The sample perceived that developments in the clinical literature superseded recommendations made by NICE. However, there was no consensus on the appropriateness of prescribing decisions outside of NICE recommendations.

**I**: *“None of this stuff [treatment decisions outside of NICE recommendations] is really very well decided or agreed, and it comes down to your clinical … feeling, really”.*

A lack of clinical evidence also influenced prescribing decisions; for example, through national drives to generate evidence on less-utilized treatments.

#### Colleagues in different hospitals

The rheumatologists expressed that differences in treatment practices between hospitals were likely. Some sought a greater understanding of these differences whereas others assigned greater value to their own personal experiences of managing patients.

**I**: *“ … what I find most helpful is to go to sessions to hear people talk about their clinical experience. Particularly people who are using drugs in different ways”.*

**D**: *“ … all that really matters to me is … knowing that what I do works for my patients”.*

The awareness of different approaches to treatment between rheumatology units appeared to facilitate informal comparisons in best-practice.

**A**: *“ … we could actually bring drug spend up significantly by doing what many people do, which is … don’t use combinations, use small-dose methotrexate, don’t use high-dose subcutaneous methotrexate … then put loads of people on biologics”.*

#### Pharmaceutical industry

Some concern was expressed that the pharmaceutical industry may have exerted influence to promote treatment decisions towards biologic therapies. One suggested advantage of prescriptive treatment recommendations was to minimize the influence of the pharmaceutical industry on routine prescribing decisions. The sample speculated that rheumatology nurse specialists, often involved in the provision of information to patients regarding biologic agents, may be susceptible to the marketing messages of pharmaceutical companies.

**E**: *“ … one of the things I’m always a little bit concerned about is … drug reps speaking to us, speaking to nurses … Because so often … you ask [the patient] to see the specialist nurse who can speak about anti-TNF treatment with them … That decision making can be influenced”.*

### Internal hospital influences

Three influences identified from the interview transcripts that were categorized as internal to a hospital are now described.

#### Systems to promote compliance with NICE recommendations

The rheumatologists described hospital-level systems, often implemented by pressure from their commissioners, to promote compliance with NICE recommendations.

**F**: *“Our CCG have imposed that on us … they actually set an ambitious target of ninety-five percent adherence to NICE”.*

Internal audits of practice were one mechanism to ensure prescribing decisions remained within the bounds of NICE recommendations; however, the frequency of audits was variable across the sample. Computerized systems that monitored prescribing decisions were perceived by some rheumatologists as a means to enforce NICE recommendations, whereas others considered these systems to be fallible.

**K**: *“ … we’ve got the discipline of the computerized prescribing system”.*

**A**: *“[The computerized prescribing system] logs if they’ve not responded to things, and you can … duck and dive a bit there”.*

#### Internal treatment pathways

Most rheumatologists explained how their prescribing decisions were, or were soon to be, guided by an internal treatment pathway that may have incorporated minor deviations from recommendations made by NICE.

**I**: *“ … we have our own pathway which … is basically the NICE pathway but there’s one or two minor exceptions”.*

#### Hospital culture

Treatment of RA was reported to have become more aggressive in recent years. However, some participants referred to ‘aggressive treatment’ in terms of rapid escalation to biologic therapy; others referred to ‘aggressive treatment’ in terms of early arthritis clinics to delay biologic therapy. Divergent views were presented across the sample on the influence that rheumatology nurse specialists had on treatment decisions (from an additional enforcer of NICE recommendations to a more passive role).

**F**: *“ … if our biologics nurse has received referrals with a DAS below 5.1, for example … they’d just bat that straight back to … the lead consultant for that patient”.*

**B**: *“Patients become very laissez-faire about being on their biologics … and I think the nurses [are the same]. As we have more and more experience, [the nurses say] ‘ah, they’ve very safe’ and they can just … put everyone on them”.*

Capacity restrictions within a hospital may have limited the rheumatologists’ ability to treat patients as they would have liked.

**K**: *“We’ve got four rheumatologists, we could probably do with a fifth. There’s been a capacity issue which has meant that … the behavior of the unit has been a bit suboptimal”.*

### Individual-level influences

Four influences identified from the interview transcripts that were categorized at the individual-level are now described.

#### Patient influence

The extent that patients could influence prescribing decisions varied across the sample. The rheumatologists described three different approaches to patient influence in the choice of anti-TNF: (i) complete freedom to choose any anti-TNF; (ii) freedom to choose an anti-TNF from a subset of agents; and (iii) no ability to choose an anti-TNF.

**C**: *“If everything else is fine … we’re happy to go with … whatever [anti-TNF] the patient wants”.*

**K**: *“For rheumatoid at the moment … we don’t really give them a choice [of anti-TNF]”.*

The rheumatologists expressed some concern that patients may modify their treatment regime without making it known during a consultation.

**A**: *“ … the other problem is that when they’re doing well on the biologics, they [patients] wind down their other treatments … the methotrexate et cetera”.*

Some rheumatologists were skeptical over patients’ abilities to make informed treatment decisions.

**D**: *“ … you know what it’s like with patients. Even if somebody changes the color of their paracetamol, they’re convinced it isn’t working as well”.*

**F**: *“ … we don’t give them options of five agents … you don’t want to bewilder patients”.*

The rheumatologists described that patients typically expressed preferences over the frequency of anti-TNF injections and mode of administration. Patient influence was considered to be sacrificed when specific treatment decisions were imposed for cost-saving reasons.

**K**: *“ … we’ve compromised patient choice in the interests of the health economy”.*

#### Clinical autonomy

Clinical autonomy over treatment decisions was valued by rheumatologists in the sample.

**I**: *“ … my view would be … to have every agent available first-line and then second-line … and third-line and, being really greedy, then have a fourth-line option which currently we don’t have … I think that’s partly why we don’t move away from anti-TNF as our first-line, because it gives us more options in the pathway”.*

Individual funding requests (IFRs) was one mechanism to facilitate clinical autonomy by obtaining approval from commissioners to prescribe treatments outside of NICE recommendations. However, the use of IFRs was variable across the sample.

**H**: *“Quite a lot [of IFRs have been undertaken] … in our area, we have actually never had a problem … I cannot recollect a single occasion that we were refused funding”.*

**D**: *“ … it takes an absolute year of paperwork to get them [IFRs], because you’ve got to go through hundreds of different committees … so I’m very glad I haven’t needed to”.*

Successful IFRs could be used as templates for future IFRs. Other rheumatologists found that changing their hospital’s internal treatment recommendations was more effective to achieve clinical autonomy.

**F**: *“ … these things come up over and over again, so you’ve got a kind of ‘Situation X IFR’ that you can use, cut and paste”.*

**I**: *“ … we never get anywhere with IFRs … In the end, after a lot of wrangling, got [a treatment outside of NICE recommendations] though as a … change to the pathway … IFRs for rheumatoid just don’t wash ‘cos it’s not an individual fight”.*

#### Consultant experience

The rheumatologists reported that their previous experiences of anti-TNF therapies influenced their decision-making. For example, older anti-TNFs were perceived positively because they had been available the longest. Negative experiences, such as a case of infection, were found to dissuade rheumatologists from using that treatment in the future.

**J**: *“ … we tend to just use what we’re familiar with, and the ones [anti-TNF agents] that have been around the longest”.*

**A**: *“We don’t use a lot of leflunomide in combination with biologics because … we’ve seen more infections. But that’s only in small numbers, but it does influence you”.*

#### Perceptions of DAS28

NICE recommendations specify that the DAS28 [33] is used as part of the criteria to determine a patient’s eligibility for anti-TNF therapy. The specific criteria for eligibility at the time of the interviews was to have a DAS28 assessment of at least 5.1 on two occasions one month apart [34]. The strict use of DAS28 assessments to determine eligibility for anti-TNF therapy was generally perceived negatively by the rheumatologists. In particular, cases where the DAS28 underestimated disease activity due to low inflammatory markers or low patient self-reporting on the visual analogue scale (VAS) were reported in the sample.

**F**: *“ … there are a few patients who we will put for anti-TNF even if their DAS is below 5.1 if … they’re ‘copers’ or ‘habituators’ … where we … from our … expert judgement feel that their disease is far more active than perhaps their VAS would indicate”.*

**A**: *“ … then there’s some people who don’t put up their inflammation tests, their ESR/CRP, and that is quite a big part of the composite [DAS28] score. So there are a lot of people who you think, ‘if only their inflammation test went up’”.*

Some rheumatologists argued that patients suffered because the DAS28 threshold recommended by NICE for anti-TNF eligibility was too high.

**J**: *“ … we have got … quite a lot of patients [with a DAS28<5.1] … They smolder in modest, moderate disease activity … probably slowly damaging their joints”.*

Table 3 reports strategies to manipulate the DAS28 assessment, described by rheumatologists in the sample, to enable more patients to receive anti-TNF therapy. For example, a single DAS28 assessment could be performed, instead of the two assessments required by the NICE eligibility criteria, to (i) reduce the time to prescribing an anti-TNF and/or (ii) avoid the risk of the second DAS28 assessment being less than 5.1. One rheumatologist expressed concern that inaccurate DAS28 scores may have been recorded within data collected for national patient registers.

**A**: *“ … I think most people lie actually [about DAS28 scores] … most people make it up … the problem for … the registry is that people make up the numbers … to keep the CCG happy … but then give those spurious numbers to the registry”.*

**Table 3:** Strategies to Manipulate the DAS28 Assessment Reported by the Sample

Number	Strategy to Manipulate the DAS28 Assessment
1	Measure disease activity using a different instrument (such as RAPID3) and convert to DAS28 scores.
2	Claim the patient has psoriatic arthritis because fewer active joints are required to prescribe anti-TNF therapy compared with RA.
3	Only perform one DAS28 assessment.
4	Stop a patient’s steroids to increase their DAS28 score.
5	Perform a DAS28 assessment when the patient has a flare in disease activity.
6	Increase the frequency of DAS28 assessments to increase the likelihood of measuring two scores greater than 5.1.

Note: The criteria by NICE to determine eligibility for anti-TNF therapy was to have two DAS28 assessments of at least 5.1 one month apart [34]. DAS28: Disease Activity Score-28 Joints; RAPID3: Routine Assessment of Patient Index Data 3

## Discussion

This study identified thirteen factors that influenced anti-TNF prescribing decisions for RA using thematic framework analysis of semi-structured interviews with rheumatologists. Factors were categorized by three primary themes: *External Environment Influences*, *Internal Hospital Influences*, and *Individual-level influences*. Awareness of these influences may contribute to understanding regional variation in treatment and the uptake of clinical recommendations in routine practice.

One conflict between participants in this study was the extent to which NICE recommendations were interpreted as flexible (advisory) or inflexible (mandatory). In 2004, Sheldon and colleagues [35] analyzed routinely collected data, patient case notes, and interviews (with leads of clinical specialties, governance, and chief executives) to evaluate the implementation of eleven exemplars of NICE guidance. Participants in the Sheldon et al. study also exhibited divergent beliefs on the purpose of NICE recommendations (advisory/mandatory) and the frequency of clinical audits. The extent to which the cost of treatment was described to influence the rheumatologists’ routine prescribing decisions also conflicted between the participants. At one extreme, cost was said not to influence routine prescribing decisions to the advantage of the patient with RA who will receive treatment; at the other extreme, cost was explicitly considered in order to sustain resources for other patients being treated elsewhere in the health care system. NICE recommend the lowest-cost anti-TNF for people with RA who meet the eligibility criteria [5]. Failure to account for the cost of prescribing decisions, which reduces the resources available for patients to benefit elsewhere in the health care system (the opportunity cost) [36], may lead to a net loss of health where the health gained by identified patients is less than the health forgone by unidentified patients [37, 38].

The rheumatologists who perceived their commissioners as an enforcer of NICE recommendations also had a tendency to describe stricter systems to ensure compliance with those recommendations at the hospital-level and fewer opportunities for patients to be involved in prescribing decisions at the individual-level. The proportion of CCGs reporting budget deficits increased since conducting this study (2015/16: 15%; 2016/17: 46%) [39]. Subsequently, the prevalence of CCG-imposed prescribing decisions, as reported by the sample, may also increase in the future. As a consequence, clinician autonomy and patient involvement in decision-making may be forfeited further, illustrating a perceived conflict between population-level treatment recommendations and the autonomy of making treatment decisions for individual patients.

Kalkan and colleagues [23] found that similar factors (clinical evidence; colleagues; departmental culture; budget constraints) influenced senior rheumatologists’ prescribing decisions with biologic agents in Sweden. Kee and colleagues [24] reported that consultants in Ireland may also strategically manipulate DAS28 assessments when considering continuation of infliximab for people with RA, if symptoms were more severe than their DAS28 score indicated. Subsequent quantitative analyses of patient-level data may be subject to bias if these observations comprise inaccurate DAS28 scores.

The small sample could be perceived as a limitation of this study; however, a larger sample is neither necessary or sufficient to obtain an understanding of a phenomenon being researched [40]. The rate of recruitment (24% of the sampling frame) was comparable to the proportion of Swedish rheumatologists recruited to the qualitative study by Kalkan et al. [23]. A second potential limitation was that this study explored factors that influenced prescribing decisions from the viewpoint of rheumatologists only. Future research could explore whether the factors that influenced anti-TNF prescribing decisions in this study are consistent across different stakeholders involved in the decision-making process, such as commissioners of health care or patients. The decision to purposefully sample rheumatologists with extensive experience of using anti-TNF agents may have masked the factors that influence rheumatologists from different practice settings or who have lower levels of experience. Future research could investigate whether the external environmental and internal hospital factors influence junior rheumatologists to the same extent that their senior colleagues have described. The sample of rheumatologists from England may also be perceived as a limitation when generalizing the results to other health care jurisdictions; however, whilst qualitative research is context-specific, the use of published clinical recommendations, the financial challenges associated with prescribing relatively high-cost biologic agents, and the existence of regional variation in care for people with RA are common features of publicly-funded and private health care systems around the world.

## Conclusions

Factors that influence routine prescribing decisions are multifaceted and may encourage divergence from published clinical recommendations. Actions that demonstrated conflict between clinical autonomy, to benefit identifiable patients, and population-level recommendations, to improve the relative cost-effectiveness of health care, were described. These influences may contribute to understanding variation in clinical practice for RA that has been reported previously.

## Data Availability

The datasets analyzed during the current study are not publicly available for reasons of maintaining participant confidentiality.

## Abbreviations

Anti-TNF: Anti-tumor necrosis factor alpha
CCG: Clinical Commissioning Group
cDMARDs: Conventional disease-modifying antirheumatic drugs
DAS28: Disease Activity Score-28 Joint Count
IFR: Individual funding request
NICE: National Institute for Health and Care Excellence
RA: Rheumatoid arthritis
VAS: Visual analogue scale
